# The Light and the Dark Side of Maternal PKU: Single-Centre Experience of Dietary Management and Emergency Treatment Protocol of Unplanned Pregnancies

**DOI:** 10.3390/nu17061048

**Published:** 2025-03-17

**Authors:** Claudia Gautiero, Iris Scala, Giulia Esposito, Maria Rosaria Coppola, Nunzia Cacciapuoti, Mariagrazia Fisco, Margherita Ruoppolo, Pietro Strisciuglio, Giancarlo Parenti, Bruna Guida

**Affiliations:** 1 Physiology Nutrition Unit, Department of Clinical Medicine and Surgery, Federico II University of Naples, 80131 Naples, Italy; claudia.gautiero@gmail.com (C.G.); giuliaesposito3107@gmail.com (G.E.); coppolamr.nutrizione@libero.it (M.R.C.); nunzia_cacciapuoti@libero.it (N.C.); bguida@unina.it (B.G.); 2 Department of Maternal and Child Health, Federico II University Hospital, 80131 Naples, Italy; 3 CEINGE-Advanced Biotechnology, Franco Salvatore, Department of Molecular Medicine and Medical Biotechnology, Federico II University of Naples, 80131 Naples, Italy; fisco@ceinge.unina.it (M.F.); margherita.ruoppolo@unina.it (M.R.); 4 Department of Molecular Medicine and Medical Biotechnology, Federico II University of Naples, 80131 Naples, Italy; 5 Department of Translational Medicine, Federico II University of Naples, 80131 Naples, Italy; pietro.strisciuglio@unina.it (P.S.); parenti@unina.it (G.P.)

**Keywords:** phenylketonuria, PKU, phenylalanine, pregnancy, maternal phenylketonuria syndrome, dietary therapy, emergency treatment, unplanned pregnancies, diet

## Abstract

**Background/Objectives**. Maternal phenylketonuria syndrome (MPKUS) is the most serious pregnancy complication of women with phenylketonuria (PKU). High phenylalanine (Phe) levels are indeed embryotoxic for the fetus. A low-Phe diet started before conception and maintained throughout pregnancy ensures optimal blood Phe concentrations (120–360 μmol/L) and pregnancy outcome. Women with unplanned pregnancies are at higher risk of MPKUS and require a rapid and sustained reduction of blood Phe. In this retrospective study, we evaluated the effects of dietary intervention on Phe levels and on the clinical parameters of offspring at birth in a group of patients with PKU. We also describe the fetal outcome of unplanned and untreated mothers with PKU. **Methods**. The cohort consisted of 13 patients for a total of 22 pregnancies: 16 successful pregnancies and 6 abortions. Pregnancies were divided into three groups: “Planned Pregnancies, PP (*n* = 5)”, “Unplanned Pregnancies, UP (*n* = 6)”, and “Unplanned and untreated Pregnancies UT (*n* = 5)”. **Results**. Women in the UP group showed higher levels of Phe than women in the PP group, especially during the first trimester. The offspring of the UP group showed no congenital malformations but lower median auxologic parameters at birth compared to those from the PP group, although these were not significantly different. The women in the UT group received the diagnosis of PKU after the birth of offspring with MPKUS. **Conclusions**. A low-Phe diet is critical to prevent MPKUS, especially when started before conception or no later than the 10th week of gestation. Intensive effort is necessary to avoid unplanned pregnancies and to identify undiagnosed women with PKU at risk of MPKUS.

## 1. Introduction

Phenylketonuria (PKU) is an inborn error of metabolism due to the deficiency of the hepatic enzyme phenylalanine hydroxylase (PAH) (MIM #261600). The condition is precociously identified by neonatal screening that in Italy has been mandatory since 1992, although regional pilot programs started earlier in some areas. Hence, a number of women successfully treated from the newborn period are now in their reproductive age.

Based on the available data, it is unclear whether high phenylalanine (Phe) levels make conception more difficult; on the contrary, studies show that hyperphenylalaninemia increases the risk of miscarriage [1,2,3]. Regardless of PKU, every pregnancy starts with a 3–5% chance of having a child with a birth defect, in proportion to the age of the pregnant woman. This is called “background risk.” Pregnant women with PKU who are on a low-protein diet with optimal metabolic control are no more likely to have offspring with a birth defect and/or intellectual disability than women without PKU [4]. The possibility of unfavorable outcome increases exponentially in pregnant women with high Phe levels [1]. Indeed, the placenta does not protect the unborn child from maternal hyperphenylalaninemia. On the contrary, there is an active transplacental transport of Phe with a mean feto-maternal gradient of 1.48 at the delivery of PKU pregnancies [4]. During pregnancy, elevated levels of Phe cross the placenta and exert teratogenic effects on the fetus, creating a condition of hyperphenylalaninemia-induced multimalformative embryofetopathy also known as maternal phenylketonuria syndrome (MPKUS). First described by Dent [5] and Mabry et al. [6], MPKUS is characterized by low birth weight (40%), microcephaly (73%), congenital heart diseases (12%), facial dysmorphism, cognitive impairment, and behavioral abnormalities (92%) [7]. The data in the literature show that 95% of mothers with Phe concentrations above 1200 μmol/L have a high probability of giving birth to children with intellectual disabilities [7]. The risk decreases as Phe levels decrease [8,9]. Maternal dietary therapy designed according to the individual Phe tolerance and started before conception is the mainstay approach for preventing embryofetopathy [8,9,10,11,12,13]. Both USA and European guidelines recommend maintaining blood Phe levels consistently within the range of 120–360 μmol/L before and during pregnancies [14,15]. These concentrations promote normal psychomotor, physical, and brain development of the offspring, especially when optimal metabolic control is reached before conception [16] and no later than the 8–10th gestational age (GA) [8,11,17,18,19]. Current European guidelines [20] also provide guidance for the clinical and nutritional management of pregnancies of women with PKU and recommend strict and individualized monitoring of energy intake, protein substitutes, maternal weight, and Phe tolerance, especially in the second and third trimester, when tolerance increases. Unlike planned pregnancies, the management of unplanned PKU pregnancies is still challenging and requires emergency measures to achieve a rapid reduction of blood Phe, ideally within 7 days [20,21]. In this retrospective study, we describe the clinical outcome of 22 pregnancies of women with PKU, the differences in the metabolic control and offspring outcome at birth between planned and unplanned pregnancies, and the Center’s standard operating procedure developed for the management of planned and unplanned pregnancies. We also describe pregnancies with MPKUS of women with undiagnosed PKU or lost to follow-up and emphasize the need for intensive efforts to avoid unplanned pregnancies and to identify women with undiagnosed PKU at risk for MPKUS.

## 2. Materials and Methods

### 2.1. Study Sample

This study involved physicians with expertise in Food Science and Inherited Metabolic Disorders and was conducted on thirteen women with PKU attending the outpatient clinic of the Physiology Nutrition Unit and of the Inborn Errors of Metabolism Unit of the Federico II University of Naples for a total of 22 pregnancies managed from 2006 to 2023. Data collection was performed by retrieving information from the patients’ medical records. The same person (C.G.) performed data recording. Five patients were secondipara. To assess pregnancy outcomes, anthropometric parameters of the infants and information on birth complications, birth defects, and intrauterine development were collected. The characteristics of the study sample are described in Table 1. Patients were classified according to untreated Phe levels and Phe tolerance into classic PKU (cPKU; Phe > 1200 μmol/L; tolerance ≤ 350 mg/day), moderate PKU (moPKU; Phe 900–1200 μmol/L; tolerance 350–400 mg/day), mild PKU (mPKU; Phe 600–900 μmol/L; tolerance 400–600 mg/day), and mild HPA (Phe 360–600 μmol/L; tolerance > 600 mg/day), as described elsewhere [22]. The study sample consisted of 8 women with classic PKU, 3 women with mild PKU, and 2 with mild hyperphenylalaninemia requiring dietary protein restriction. The historical tolerance varied in the range of 340–2100 mg/day. The mean age of the pregnant women at conception was 30.2 years (range 24–39 years).

**Figure 1:**
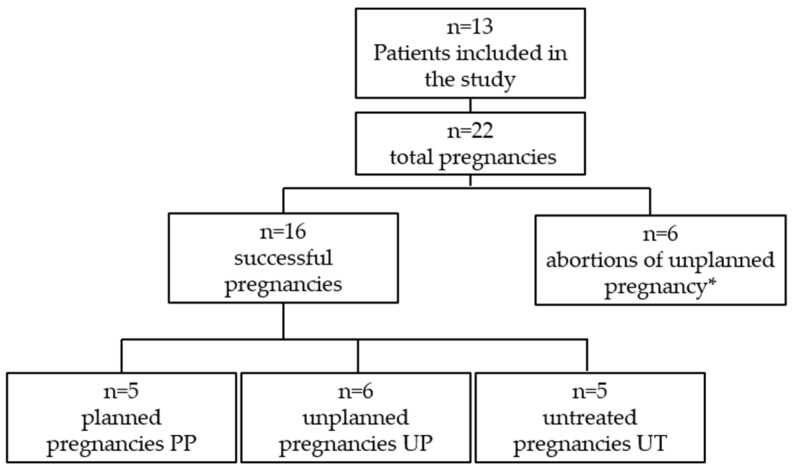
Pregnancies included in this study. Group PP: planned pregnancies; group UP: unplanned pregnancies; group UT: unplanned and untreated pregnancies. * Three women who had abortions also carried a pregnancy to term; one woman had two therapeutic abortions and one successful pregnancy.

The pregnancies included in this study and the numbers by groups are shown in Figure 1.

Successful pregnancies were divided into three groups: “Planned Pregnancies, PP (n = 5)”, “Unplanned Pregnancies, UP (n = 6)”, and “Untreated Pregnancies, UT (n = 5)”. The PP group included both women with PKU adherent or partially adherent to the prescribed diet before pregnancy planning and women off-diet who came to the Clinic to plan their pregnancy. The adopted standard operating procedures for the management of the preconception and pregnancy periods in women with PP is reported in Supplementary File S1.

In the preconception phase, women were advised to use active contraceptive methods. When plasma Phe reached stable values in the target range for three consecutive measurements, they were allowed to stop using contraceptive methods. The desired Phe target was 120–360 μmol/L; however, women were encouraged to achieve Phe values between 120–240 μmol/L.

The UP group included all unplanned pregnancies of women that came to the Clinic after conception. The emergency protocol adopted by the Clinical Centre is described in Supplementary File S2.

In both PP and UP, standard operating procedures were further adapted to the patient’s needs. Family counseling was provided by the medical team; psychological support for the woman and her family was recommended at the Psychology Unit of our Hospital but the psychologist was not present in our metabolic team.

The UT group included unplanned and untreated pregnancies during all three trimesters of pregnancy. The patients were off-diet with absent metabolic control. Patients in the UT group were unaware of having PKU that was diagnosed after the birth of a child affected by MPKUS. The group of unsuccessful pregnancies included women who underwent one or more therapeutic abortions or women who decided to voluntarily terminate their pregnancy. Participants received both oral and written information about the study and signed the data consent sheet before inclusion. The Institutional Ethical Committee (protocol n. 30/15/ES1) approved this study.

### 2.2. Amino Acid Assay

AA analysis was performed using patients’ serum samples collected in the morning after a fasting period of 8–10 h. Aliquots of serum samples were processed by high-performance liquid chromatography (HPLC) and amino acid contents were measured by an Agilent Technologies 1200 Series LC System using an Agilent Zorbax Eclipse XDB-C18, Agilent, Santa Clara, CA, USA analytical column (5 μm, 4.6 × 150 mm) and Agilent Eclipse XDB-C18 analytical guard column (5 μm, 4.6 × 12.5 mm). Metabolite derivatization was performed in automated mode using o-phthalaldehyde (OPA) and 9-fluorenylmethyl chloroformate (FMOC) for primary and secondary amino acids, respectively. The chromatographic separation was carried out using 40 mM phosphate buffer pH 7.8 as solvent A and CH_3_CN/CH_3_OH/H_2_O (40/40/20) as solvent B. The flow rate was set at 1.3 mL/min and temperature at 40 °C. The linear gradient was the following: from 10% to 20% of solvent B in 6 min, from 20% to 27% of solvent B in 6 min, from 27% to 60% of solvent B in 10 min, from 60% to 100% of solvent B in 2 min plus an isocratic step to 100% of solvent B over 6 min. The single amino acids were identified according to their retention time and quantified to compare absorption in respect to standard compounds in the calibration solution, a mixture of 200 μM of amino acids.

### 2.3. Anthropometric Parameters

During the gestation period, anthropometric parameters were collected weekly. Anthropometric assessments of nutritional status included weight, height, and body mass index (BMI in kg/m^2^). Body weight was measured to the nearest 0.1 kg using a mechanical scale (SECA). GA expressed in weeks was considered. Anthropometric parameters of the offspring at birth were collected from medical records provided by the patients. The following offspring parameters were collected for this study: weight and length, head circumference, and Apgar index. Weight, length, and occipitofrontal circumference percentiles were calculated considering sex, GA, and presence or absence of first-born children [23].

### 2.4. Dietary Management During Pregnancy

The setting of a proper dietary regimen considered the initial blood level of Phe, individual tolerance, the patient’s age, and her nutrient requirements. The individualized dietary intervention included: restriction in the intake of natural proteins, especially meat, eggs, fish, cheese, preferring a diet on a vegetarian imprint; the use of low-protein products; supplementation with amino acid mixtures with reduced Phe content, enriched with vitamins, minerals, and essential fatty acids. Women with PKU consumed low-protein commercial foods (cookies, flour, pasta, bread), combined with Phe-free amino acid medical formulas provided free of charge by the Italian Health System. Full details of the dietary management are available in Supplementary File S3.

### 2.5. Statistical Analysis

Statistical analysis was performed using SPSS 29.0 software. The Mann–Whitney U-test was used for non-normally distributed datasets, expressed as median and interquartile range (IQR). In the case of normal data distribution, results were expressed as mean ± standard deviation (SD) and significance was assessed with the Student’s *t*-test. Significance was assumed for *p* < 0.05. Phe levels at T0 (preconception period), T1 (first trimester), T2 (second trimester), and T3 (third trimester) were considered, and the medians with IQR at each trimester were calculated.

## 3. Results

### 3.1. Phenylalanine and Tyrosine Levels and Phe/Tyr Ratio (PP and UP Groups)

In the five planned pregnancies (PP), patients had started dietary therapy and monitoring of Phe levels before conception. The duration of the preparation phase varied widely among the patients. The low-Phe diet resulted in the reduction of Phe levels in all patients as early as the first follow-up. The difficulty was to stabilize Phe levels in the optimal range for at least three consecutive measurements and thereafter maintain the result until conception. This was especially the case for women who could not conceive over a short period who struggled between their motherhood desire and the frustration from not getting pregnant. For this reason, the duration of the preparation phase and the stabilization of Phe levels in the PP group was different among patients (mean SD: 4.6 ± 3.2 months; range 2–9 months). Patients who had planned pregnancy started their gestation with Phe levels between 120 and 240 μmol/L, with the exception of one patient whose first Phe value after conception was 75.6 μmol/L (median, IQR: 180 μmol/L, 80.4–180 μmol/L; range 75.6–220.2 μmol/L). Plasma Phe remained in the optimal range throughout the pregnancy, with the exception of a few outliers above the range due to intercurrent infections and a few low Phe values promptly corrected with dietary intervention.

Six women had unplanned pregnancies (UP) and came to the Clinic between the 4th and the 10th GA (mean 7.2 ± 2.0 week). Five of the six UPs had high Phe levels (median, IQR: 744 μmol/L, 708–900 μmol/L; range 558–1002 μmol/L) at the first outpatient visit at which they were found to be already pregnant. Dietary treatment was started within 24 h from pregnancy notification according to the Center’s SOP. Target Phe values were reached between the second and third week of the diet. One of the six UPs started pregnancy with in-target Phe values (310 μmol/L). The pregnant woman came to our observation at the 8th GA, when she started dietary therapy; the patient had mild PKU and was on sapropterin therapy and off-diet. After a multidisciplinary nutritional and genetic consultation, the woman decided to discontinue pharmacological therapy and start a low-Phe diet as at that time there were no sufficient data on the safety of sapropterin during pregnancy. The new diet was calculated according to the known Phe tolerance before sapropterin. The woman showed a good adherence to the nutritional scheme and her Phe values remained in the desirable range throughout the pregnancy (median, IQR: 300 μmol/L, 225–312 μmol/L). One patient in the UP group never reached the recommended Phe concentrations during her pregnancy, as she continued to consume not-admitted foods despite continuous advice from our medical team and her family. Informed of the MPKUS, the patient decided to carry the pregnancy to term.

**Figure 2:**
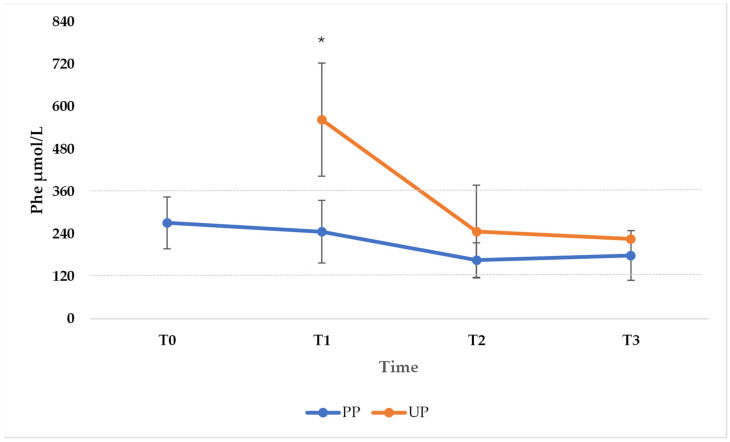
Phe levels of women in the “Planned Pregnancies PP (n = 5)” vs. “Unplanned Pregnancies, UP (n = 6)” group. The points in the graph represent the mean of the medians of Phe values at T0, T1, T2, and T3 in the PP group and at T1, T2, and T3 in the UP group. Values were higher in women in the UP group throughout pregnancy; in the first trimester, the difference was statistically significant. * *p* < 0.05. Test T-student PP vs. UP (T1 *p* = 0.002; T2 *p* = 0.31; T3 *p* = 0.49). Dotted lines: current recommended level of phenylalanine during pregnancy between 120–360 µmol/L.

Compared with women in the PP group, women in the UP group showed higher Phe values throughout pregnancy and, in the first trimester, this difference was statistically significant (Figure 2). 

It can also be seen from the standard deviations that the fluctuations in values were larger in the UP group than in the PP group, especially in T1 (PP group: mean and SD at T0 271.6 ± 73.3; mean and SD at T1 246.5 ± 88.5; mean and SD at T2 166.1 ± 48.6; mean and SD at T3 179.0 ± 70.3. UP group: mean and SD at T1 563.3 ± 144.2; mean and SD at T2 246.6 ± 88.5; mean and SD at T3 225.9 ± 131.1). The Mann–Whitney test was performed on both minimum and maximum values of Phe in the first (T1), second (T2), and third (T3) trimesters. Special attention was paid to the maximum levels achieved during the three trimesters of pregnancy as it was of fundamental importance that they were kept in the optimal range for the prevention of MPKUS (Table 2).

In some cases, Phe values were below 120 μmol/L and Phe intake was promptly recalculated. In addition to Phe values, Tyr values and Phe/Tyr ratio in the PP and UP groups were also compared. Tyr levels were above 30 µmol/L in all women (median, IQR: 46, 40–52.3; range 32–156 µmol/L; reference values 45–107 µmol/L). The Phe/Tyr ratios were as follows: median, IQR: 3.23, 2.20–5.13; range 0.69–26.4. No clinically significant change of amino acids other than Phe and Tyr were observed.

### 3.2. Anthropometric Characteristics of Women

**Figure 3:**
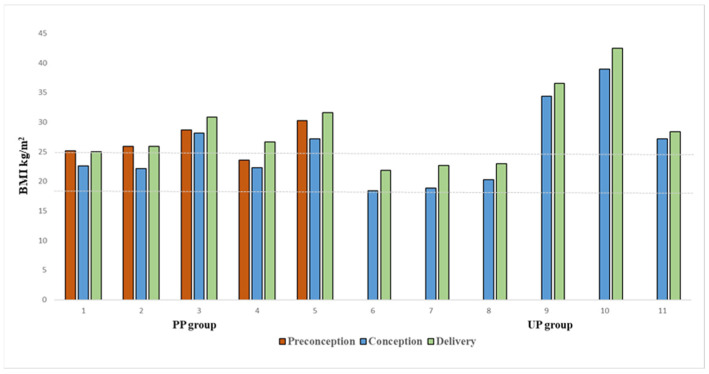
Time trend of BMI in individual pregnancies (11 pregnancies; PP group = 5 pregnancies, UP group = 6 pregnancies) from the preconception period to delivery. Dotted lines: desired BMI between 18.5 and 24.9 kg/m^2^.

Before starting dietary treatment for pregnancy planning, women in the PP group were all overweight but for one (mean and SD of BMI 25.9 ± 2.1 kg/m^2^; range 23.6–28.7 kg/m^2^). During the preparation phase (T0), patients started specific dietary therapy, also aimed at achieving an ideal BMI to start pregnancy, losing an average of 5.56 ± 3.24 kg. All but two of the overweight patients reached a preconception normal weight status (mean and SD of BMI 23.9 ± 2.9 kg/m^2^), but they all lost weight. In the UP group, three out of six patients were overweight, but, despite this, they did not have a pre-pregnancy diet; the dietary pattern assigned after conception still allowed them to keep their weight under control but not to start the pregnancy at an ideal weight. Both the patients in the PP group and in the UP group received their specific diets based on requirements during the three trimesters of pregnancy, which was assigned not only to keep Phe levels under control but also to avoid excessive and unnecessary weight gain in pregnancy. Desirable weight gain was always assessed on the basis of baseline BMI. In both groups, weight gain was similar and was kept under control until the end of pregnancy (mean and SD 8.0 ± 2.6 kg; range 5.8–11) (Figure 3), and this emphasizes the importance of nutrition in pregnancy.

### 3.3. Dietary Therapy

Management of the low-Phe diet is very challenging, which is why most patients were not fully adherent to the dietary pattern during adolescence and early adulthood. Protein intake was calculated based on patients’ body weight and increased at each trimester regardless of BMI. Daily protein averaged 72.50 ± 9.2 g/day in the first trimester (range from 60–91 g/day), 77.00 ± 10.2 g/day in the second trimester (range from 66–97 g/day), and 87.25 ± 10.5 g/day in the third trimester (range from 70–107 g/day). Daily protein intake was divided into natural protein and medical formula. Natural protein intake depended on patients’ tolerance and genotype. In women with classic PKU and mild PKU, on average 86% of the daily protein requirement was provided with medical formula, and only about 14% of the total protein were natural proteins. In women with mild HPA, about 38% of the protein requirement was provided with medical formula, while about 62% was taken through food.

### 3.4. Phenylalanine Tolerance

Phe tolerance generally increases during pregnancy [24], mostly depending on residual PAH activity. In our cohort, tolerance did not vary during the three trimesters in women with classic PKU (mean, SD: 372 mg/day, 109.02), in women with mild PKU (mean, SD: 652.5 mg/day, 294.09), and, unexpectedly, also in women with mild HPA (mean, SD: 1725 mg/day, 735.06). In only one patient, we noticed an increase in tolerance from the first to the third trimester (Phe T0 460 mg/day, Phe T1 760 mg/day, Phe T3 1090 mg/day); the patient was suffering from mild PKU. No differences in tolerance were found in secondipa women during different pregnancies.

### 3.5. Adherence to Dietary Therapy

**Figure 4:**
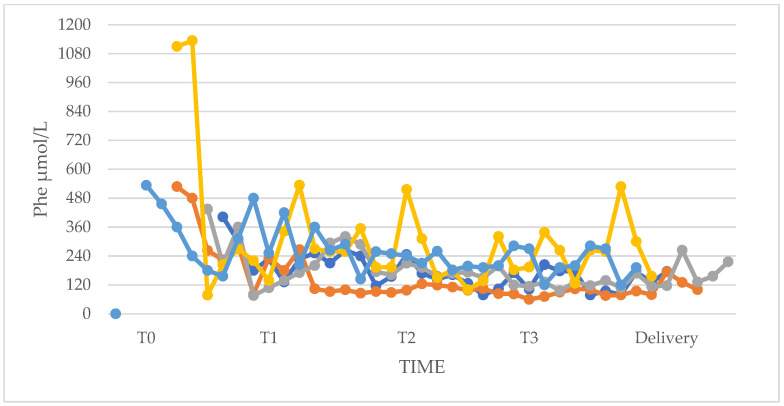
Phe levels in n = 5 pregnancies of the PP group.

In three pregnancies of the PP group, adherence to diet was optimal during the three trimesters and Phe levels stabilized at values <360 μmol/L from T1 to T3 (median, IQR: 132.0, 101.4–177.6; range 59.4–339 μmol/L). When blood Phe was < 120 μmol/L, dietary Phe was increased by 50 mg/day until Phe > 120 μmol/L was reached. In two pregnancies in the PP group, adherence to diet was suboptimal during the three trimesters with some Phe values above 360 μmol/L. In one of them, also recurrent upper respiratory tract infections and urinary tract infections caused peaks of Phe. In these two cases, Phe levels showed peaks between 420–534 μmol/L (median, IQR: 258, 192.6–306; range 100.2–534 μmol/L). Phe levels of the PP group pregnancies are shown in Figure 4.

**Figure 5:**
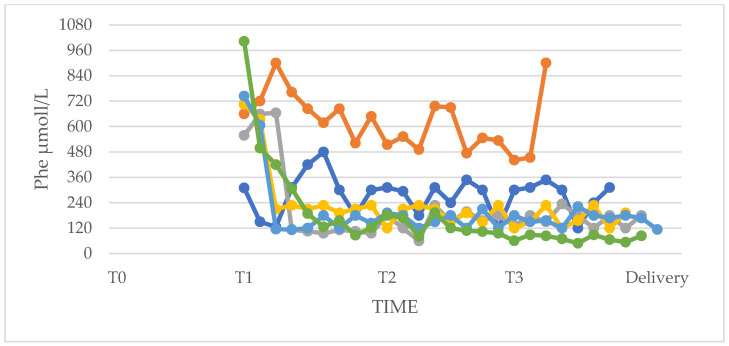
Phe levels in n = 6 pregnancies of the UP group.

In pregnancies of the UP group, the fetus was exposed to high levels of Phe at the beginning of pregnancy. In four UP pregnancies, initial Phe levels were elevated (median, IQR: 645.6, 570–696; range 420–1002 μmol/L) and fell within the recommended range in 1–3 weeks with subsequent good metabolic control and dietary adherence throughout the pregnancy. Phe levels decreased and stabilized in the desirable range until delivery (median, IQR: 150, 114.6–189.6; range 48.6–306 μmol/L). In the other two UP pregnancies, compliance was suboptimal. In one of the two pregnancies, Phe values were always above the reference threshold (Phe min 441.6; Phe max 900 μmol/L). In the second pregnancy, the patient showed Phe values in the range already at the beginning of pregnancy, with few peaks outside (median, IQR: 300, 225–312; range 120–480 μmol/L) despite not having an optimal dietary adherence (Figure 5).

### 3.6. Pregnancies of Previously Undiagnosed PKU Women (UT n = 5)

**Figure 6:**
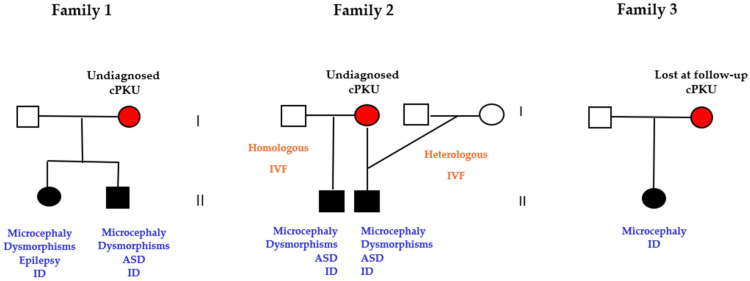
Maternal PKU syndrome (MPKUS) in offspring of women with undiagnosed PKU. Abbreviations: ID: intellectual disability, ASD: atrial septal defect.

Five pregnancies of three mothers were not treated because the women were unaware of being affected by PKU (Figure 6).

In family 1, the woman was diagnosed with classic PKU (Phe 1614 μmol/L) at 38 years old after the birth of two siblings affected by microcephaly, intellectual disability, and facial dysmorphisms after a 6-year diagnostic odyssey of her two children. The family, of Italian origin, had lived in Germany for 15 years. The woman had normal fluid intelligence (Raven matrices; IQ = 100), although she reported difficulties in concentration and logical reasoning and low self-esteem. In family 2, the parental couple conceived their first child after a homologous in vitro fertilization due to couple infertility. The male baby presented with intellectual disability, microcephaly, atrial–septal defect, and facial dysmorphisms. When the child was 7 years old, still undiagnosed, the couple decided to have a second pregnancy by a heterologous in vitro fertilization. Although genetically different from the parents, the second-born baby being a phenocopy of the first sibling, an intrauterine teratogenic effect was hypothesized. Consequently, the mother received the diagnosis of classical PKU (Phe 1584 μmol/L) and the siblings of MPKUS. At the time of the diagnosis, the woman was 44 years old. Similarly to the first family, the woman did not have intellectual disability and worked as a nurse in a dermatology unit of a small suburban hospital. The third woman (family 3) was diagnosed by neonatal screening and treated until she was 7 years old, when her parents died. At that point, she was raised by her aunt and stopped the diet and medical check-ups. Growing up as a healthy and normally performing woman and unaware of the consequences of PKU, she gave birth to her first child after an untreated pregnancy. At 3 years old, the baby started medical consultations for microcephaly and developmental delay. After a deep anamnestic interview, the mother recalled that during childhood she attended medical visits and followed a special diet; PKU was suspected and confirmed by amino acid analysis (Phe 1240 μmol/L). All 5 children had a normal amino acid profile and had the typical dysmorphic features of MPKUS.

### 3.7. Abortions of Unplanned Pregnancies

Six unplanned pregnancies were not successfully delivered. Three out of six pregnancies underwent a therapeutic abortion because of high Phe levels after positive pregnancy test of women with classical PKU with very poor dietary adherence (mean and SD 1458 ± 174 μmol/L, range 1248–1632). Ultrasound examination showed intrauterine growth retardation in all cases and a congenital heart defect in one case. A fourth pregnancy terminated with a therapeutic abortion after the diagnosis of univentricular heart and myelomeningocele in the fetus at 14 weeks of GA. In this case, the woman had an unplanned pregnancy; however, a hypoproteic diet was started at the 5th week of GA and Phe levels fell from 1219 μmol/L to 300 μmol/L in 4 days and remained well below the threshold of 360 μmol/L until termination. For the severity of the fetal malformations and the good metabolic control starting from the 5th week of GA, the malformations were considered possibly not related to MPKUS and further genetic analysis addressing differential diagnosis were suggested to the couple. In two out of six pregnancies, the decision to terminate was voluntary: one abortion was the third conception of the woman of family 1 who already had two children affected by MPKUS and felt unable to carry out a further pregnancy with the challenge of a low-Phe diet. The other woman, with mild PKU, had a voluntary abortion as she already had a first child affected by a chromosomal disorder.

### 3.8. Offspring Neonatal Characteristics

The mean and SD of GA was 37.5 ± 2.2 weeks (range 36–42). Offspring (n = 11; 7 males and 4 females) were evaluated at birth: all infants had normal Phe levels and therefore no one was diagnosed with PKU. Among the children in the UP group, one child was excluded from the statistical analysis because he had a chromosomal abnormality (16q11.2q21 duplication) not related to maternal PKU. The characteristics of the offspring of the PP and the UP group are shown in Table 3.

No children in the PP and UP groups had malformations. In the PP group, all newborns were appropriate for GA. All children had normal occipital frontal circumference (OFC) measures except one child who showed reduced head circumference (31.8 cm, 4th percentile) in the context of a familial microcephaly. Three infants in the UP group showed low birth weight and short length (3rd, 2nd, 5th percentile); in addition, two of them also showed low head circumference (2nd, 4th percentile). The mother of UP group, who was on sapropterin therapy until the discovery of the pregnancy, conceived a child with normal neonatal parameters (weight = 3.650 kg; length = 51 cm; head circumference = 36.5; Apgar index 8-8). Despite the difference in growth parameters between the PP and UP groups not being statistically significant, probably due to the small sample, the medians (and IQR) were lower in the UP newborn compared to the PP. All the children of the UT group (undiagnosed maternal PKU) were affected by MPKUS with microcephaly, growth retardation, and delayed psycho-motor development.

## 4. Discussion

Pregnancy planning is the safest method to avoid or minimize the risk of MPKUS, which can be prevented by a low-Phe diet started before conception. In this study, the data represent a retrospective collection of Phe levels, anthropometric maternal variations during pregnancy, and offspring neonatal characteristics. During pregnancy, women of the UP group showed higher Phe levels than women of the PP group, especially during the first trimester of pregnancy, where the difference was significant. For patients in the UP group, it was crucial to apply an emergency protocol to rapidly reduce Phe levels. Guidelines recommend a reduction in amino acid levels no later than the 8th–10th GA [8,13,18,19]. In our cohort of UP pregnancies, reduction of Phe levels was achieved within the 10th GA in all women and the time to target ranged from 1 to 3 weeks. The offspring of women with PKU are at high risk of developing MPKUS, an embryofetopathy caused by elevated plasma concentrations of Phe crossing the placenta during pregnancy [25]. The offspring born from UP pregnancies showed no malformations at birth due to hyperphenylalaninemia, although the characteristics at birth (growth, head circumference, and Apgar index) showed a reduced trend compared to the offspring of the PP group. Despite the difference in our population not being statistically significant, the small sample size may limit the statistical power of this study to address the difference in anthropometric measures of the offspring and we suggest paying special attention to intrauterine growth in unplanned pregnancies. All children born from planned pregnancies had an overall normal intrauterine development, with characteristics at birth appropriate for GA, showing that the occasional and transient Phe peaks observed during some pregnancies and rapidly managed by dietary changes did not affect the intrauterine development of the offspring. In one UP pregnancy, Phe levels never reached the recommended concentrations during the three trimesters, but the baby was born without malformations and with normal growth parameters. Unfortunately, we do not have information on the intellectual performance of this child because the family was lost at follow-up after delivery. Clear standard operating procedures (SOPs) and a multidisciplinary approach for the management of PP and emergency protocols for UP pregnancies are mandatory. Emergency protocols for UP are very scarce in the literature and best practice recommendations are lacking. Only a few studies report indications for the dietary and clinical management of UP [21,26] and consensus guidelines further adapted to each national health system are necessary. In our Centre, SOPs were developed on clinical experience, on the available resources of the Institution and updated according to the literature data (Supplementary Files S1 and S2). In UP pregnancies, the key work was the inclusion of the women in a multidisciplinary approach as soon as the first 24 h from pregnancy notification. The core multidisciplinary team included the nutritionist, the physician with expertise in metabolic disorders, and a geneticist. At the same time, the laboratory staff was informed to obtain Phe results within 24 h. Another key point was the inclusion of the family in all the steps of the counseling foreseen for UP pregnancies, considered at-risk pregnancies. After this first engagement, women were advised about the opportunity of a specific obstetric follow-up with the high-risk pregnancy team. In our experience, unplanned pregnancies starting with Phe values no higher than 563.3 ± 144.2 μmol/L and approached with the emergency protocol successfully completed their pregnancies. Greater problems were encountered in the case of UPs starting with Phe values over 1200 μmol/L in women off-diet. In this case, women may experience problems in rapidly adapting to the Phe-restricted regimen with dietary errors that may compromise metabolic control and fetal outcome. This was the case for three women with cPKU that proceeded with therapeutic abortion because the first trimester echographic screening revealed IUGR and/or cardiac malformations in the fetus. Beyond the Phe-restricted diet, it must be emphasized that diet is essential in all pregnancies, irrespective of PKU, because it allows the correct protein and caloric intake, an adequate fetal intrauterine growth, and lower maternal complications such as gestational diabetes, pregnancy-induced hypertension, and likelihood of caesarean section [27]. In our cohort, diet in the preconception period resulted in weight loss in overweight patients and better weight management during the three trimesters of pregnancy. Weekly check-ups were essential to assess adherence to dietary therapy and to adjust amino acid intake according to Phe fluctuations; in particular, frequent monitoring of blood Phe is essential as high amino acid values lead to teratogenesis, but at the same time, suboptimal values, when combined with reduced caloric intake, lead to an increase in blood Phe due to muscle catabolism to compensate for the low plasma concentration of this essential amino acid. Therefore, a delicate balance between caloric intake and protein reduction must be maintained to meet the needs of the pregnant patient and her fetus. Values below 120 μmol/L should also be corrected as they may increase the risk of reduced intrauterine growth (IUGR), especially in the second part of pregnancy [28]. This hypothesis is also supported by animal data [29] which showed that a low-protein diet reduces circulating essential amino acids and leads to intrauterine growth restriction. For a patient with PKU, dealing with pregnancy is not easy and the support of a medical team and of all family members is necessary. This also emphasizes the need for patients to be present at all outpatient check-ups to carefully monitor Phe levels, to analyze food diaries, to assess weight gain/loss, and to psychologically support expectant mothers. Not all patients were able to achieve a perfect adherence for all three trimesters of pregnancy, underscoring the difficulty of a reduced Phe diet. Although women are strongly encouraged to achieve and maintain Phe levels in the safe range, the expectation that no single sample would show elevated Phe concentrations throughout the entire gestational period is not realistic, considering that the patients in question are pregnant women kept on a restrictive diet in terms of food choice and that the gestational period itself is complex and characterized by hormonal changes. In addition, seasonal illnesses or pregnancy vomiting or fasting from nausea may lead to sub-optimal Phe levels in some cases [30]. The weekly multidisciplinary follow-up minimizes Phe fluctuations and overcomes those barriers. Medical formulas are a fundamental part of the Phe-restricted diet. In Italy, all medical/nutritional products are provided free of charge by the National Health System and all PKU patients can be treated without economic barriers. In our cohort, all patients showed an optimal adherence to the prescribed medical formula. An important point was the involvement of the women in the choice of the medical formula that the patient considered more palatable and with less gastrointestinal discomfort.

In our cohort, tolerance to Phe was stable in all women except one with mPKU. This observation was surprising compared to the literature data suggesting an increase of the tolerance to Phe from the second trimester onwards [24]. On the contrary, in our cohort, the increase of protein intake was mainly due to the increase of the recommended protein requirements during pregnancy trimesters, according to LARN, and mainly covered by medical products. Offspring were not affected by PKU. Unfortunately, we do not have an explanation for the discrepancy of our observation compared to the available literature. Further prospective studies could help to better clarify this aspect.

One woman of our cohort with mPKU started pregnancy on sapropterin therapy that was discontinued due to safety concerns at that time. However, in 2024 the final analysis on the efficacy and safety of sapropterin before and during pregnancy of both the European and US registries was published, pointing to good safety profiles for both the mother and the fetus, especially in the European registry [31]. In the case of insufficient metabolic control, sapropterin therapy may be considered in women responsive to sapropterin.

In our cohort, three women gave birth to five children with severe MPKUS because they were unaware of having PKU at the time of conception: in two cases, women were born before the implementation of the neonatal screening, that in Italy was made mandatory from 1992 (law 104, 5 February 1992, art 6); in one case, the woman was diagnosed by neonatal screening and then was lost at follow-up. The women of family 2 conceived by PMA in advanced reproductive age. These cases raise an important issue: in most countries, PKU newborn screening was implemented less than 40–45 years ago; hence, there may be still fertile women not screened for PKU. In Italy, as an example, women older than 33 years old may have undiagnosed PKU. This is particularly true in developing countries, where PKU screening has been implemented only in recent years or not implemented at all. Due to migration, undiagnosed PKU and MPKUS in affected children must be considered also in countries with a longer history of PKU screening. Due to medically assisted procreation techniques (PMA), infertile women may conceive at >45 years old. Also, adult women screened and treated for PKU in the first years of life may be lost at follow-up and, if the family is missing, may be unaware of the importance of dietary intervention during pregnancy to avoid MPKUS. In recent years, the literature has given dramatic examples of babies with MPKUS born to mothers with previously undiagnosed PKU [32,33,34,35]. An additional complication relies on the fact that undiagnosed PKU women may have a normal IQ. In a review published in 2008, Hanley estimated that approximately 10% of women with cPKU (Phe > 1200 μmol/L), 30–50% of women with mildPKU (600–1200 μmol/L) and 97% of women with mildHPA (200–600 μmol/L) could have normal IQ [36] and proposed mandatory and non-mandatory criteria for case finding of undiagnosed women with PKU. According to their template, the diagnosis of PKU should be considered in women with: 1. a definite or suggestive familiar history of PKU; 2. a previous offspring with idiopathic microcephaly and/or intellectual disability; 3. borderline IQ or intellectual disability 4. born before the start of PKU neonatal screening in their country; 5. offspring with congenital heart disease and/or IUGR. Based on our experience with family 2, we also suggest excluding the diagnosis of PKU in women not screened at birth who access PMA techniques. Finally, as suggested by Wiedemann and colleagues [32], undiagnosed women with PKU could be also identified among mothers of children positive to the PKU newborn screening. Although some of those measures could be considered cost- and time-consuming, any action aimed at identifying undiagnosed fertile women should be intended as primary prevention of an avoidable intellectual disability and thus should strongly be pursued.

## 5. Conclusions

Faced with the constant increase in the number of children delivered by mothers with PKU, the development of standard operating procedures and training programs for women of childbearing age is crucial. These programs should be adapted to the specific needs of each patient and should include information on maternal PKU starting from adolescence, accurate transition to adult care to avoid the risk of being lost at follow-up, engagement of the family, and psychological support. Psychological support may significantly improve stress, adherence to prescriptions and postpartum adjustments, and may also reduce the cases of lost-at follow-up after delivery. Ideally, the psychologist should be included in the multidisciplinary team and, if unavailable, women should be encouraged to undertake a psychological support program. Genetic counseling is also very important in the approach to maternal PKU to review genotype analysis, to discuss recurrence risk regarding—but not limited to—PKU and Phe levels, to reinforce education on contraception, and to update informative documents for the obstetrician. Both geneticists and gynecologists should keep in mind the importance of the targeted screening of women born prior to newborn screening legislation.

Indeed, the critical point remains the prevention of MPKUS through careful nutritional education and the need for consistent contraceptive methods in case of inadequate blood levels of Phe during pregnancy planning. It is also necessary to reinforce the information for pediatricians, family doctors, geneticists, and gynecologists–obstetricians about the characteristics of MPKUS and to screen women for the risk of MPKUS. Simple questions may help in the identification of women possibly affected by PKU: 1. Was the woman born before the start of the PKU screening program? 2. Is the woman intelligent? Did she need a school support program? 3. Is there any offspring with intellectual disability and/or microcephaly and/or malformations in the family?

Inadequate management of maternal PKU risks may compromise the success of newborn screening in public health.

## Figures and Tables

**Table 1:** Characteristics of the study sample.

Pregnant women with PKU	*n* = 13
Pregnancies (including multiple pregnancies and abortions)	*n* = 22
PKU classification	Classic PKU = 8 women; 14 pregnancies (PP = 2; UP = 2; UT = 5; AB = 5) Mild PKU = 3 women; 5 pregnancies (PP = 2; UP = 2; AB = 1) Mild HPA = 2 women; 3 pregnancies (PP = 1; UP = 2)
Historical tolerance (dietary Phe; mg/day)	Range 340–2100
Maternal age at conception (years)	Mean (SD) 30.2 ± 4.8 Range 24–39

Data are expressed as mean and standard deviation (SD). Abbreviations: PKU, phenylketonuria; HPA, hyperphenylalaninemia, PP: planned pregnancy; UP: unplanned pregnancy; UT: unplanned and untreated pregnancy, AB: abortion.

**Table 2:** Minimum and maximum median plasma Phe concentrations with interquartile range (IQR) in the PP group (*n* = 5) and in the UP group (*n* = 6). Mann–Whitney test performed on minimum and maximum Phe values at T1, T2, and T3.

Time			Median Phenylalanine Concentration (μmol/L)			
			*p*-Value			
	Median Min (IQR)		Median Max (IQR)		on Min Phe Values	on Max Phe Values
	PP	UP	PP	UP		
I trimester (T1)	132 (108–138)	354 (78–630)	324 (306–534	726 (594–864)	1	0.030
II trimester (T2)	114 (102–132)	156 (114–192)	258 (210–468)	450 (228–666)	0.329	0.429
III trimester (T3)	102 (96–126)	108 (96–114)	264 (198–342)	276 (138–582)	0.931	1

**Table 3:** Characteristics of offspring at birth in the “Planned Pregnancies PP” and “Unplanned Pregnancies UP” groups.

	Offspring PP (N = 5) Males = 1, Females = 4	Offspring UP (N = 5) Male = 5, Female = 0	*p* Value
Birth weight (Kg) Median (IQR)	2.79 (2.74–3.33)	2.40 (2.30–2.85)	0.690
Weight percentiles Median (IQR)	21 (19–74)	5 (3–10)	0.151
Length (cm) Median (IQR)	49 (47–49)	45 (45–50)	0.600
Length percentiles Median (IQR)	48 (19–56)	8 (4–21)	0.310
Head circumference (cm) Median (IQR)	33 (32.5–34)	32.5 (32–33)	0.800
Head circumference percentiles Median (IQR)	40(40–47)	23 (2–27)	0.548
Apgar index Median (IQR)	8.0 (8.0–9.0)	7.0 (7.0–8.0)	0.400

## Data Availability

The data are stored in a database at the Department of Clinical Medicine and Surgery, Nutrition Physiology Unit, University Federico II of Naples and at the Department of Maternal and Child Health, Federico II University Hospital, Naples 80131, Italy. They are available upon reasonable request.

## Supplementary Materials

The following supporting information can be downloaded at: https://www.mdpi.com/article/10.3390/nu17061048/s1, Table S1: Standard operating procedure for the management of planned pregnancies (PP) of women with PKU adopted by the Clinical Center; Table S2: Standard operating procedure for the management of unplanned pregnancies (UP) of women with PKU adopted by the Clinical Center; Table S3: Dietary macronutrient composition for patients in pregnancy preparation and during the pregnancy, based on the latest LARN [37].
