# Supplementary material for: The Light and the Dark Side of Maternal PKU: Single-Centre Experience of Dietary Management and Emergency Treatment Protocol of Unplanned Pregnancies
Source: Nutrients. 2025 Mar 17;17(6):1048. doi: 10.3390/nu17061048 (PMC11944931; doi:10.3390/nu17061048)
Supplement: Supplementary file 1 [file nutrients-17-01048-s001.zip › nutrients-3533438-supplementary.docx]

***Supplementary Materials***

**Supplementary File 1**

**Supplementary table S1**. Standard operating procedure for the management of planned pregnancies (PP) of women with PKU adopted by the Clinical Center

|  | **Assessment** | **Items analyzed/discussed** | **Note** |
| --- | --- | --- | --- |
| **Pre-conception period** | Medical  evaluation | Physical examination  Provide information on the type and timing of check-ups during the preconception period and during pregnancy  Review pharmacological therapies for PKU (sapropterin, pegvaliase)  Provide information on the use of pharmacological therapies during pregnancy | Every 3 months  At least once |
|  | Nutritional evaluation | Weigh, Height, BMI, Body composition  Review Food diary  Review Tolerance to Phe  Review lifestyle (e.g., physical activity)  Review acceptance of medical formula (in terms of palatability and personal choice)  Review availability of hypoproteic foods and medical formula  Reinforce information on the type and timing of check-ups during the preconception period and during pregnancy | 1/month |
|  | Genetic  counseling | Review genotype analysis  Preconception counseling of the couple  Counselling on recurrence risk  Information of the partner about PKU and risk of MPKUS  Information on the possibility for the partner to perform the mutational analysis of the PAH gene to assess his eventual carrier status.  Information on the neonatal screening for PKU for all newborns in Italy  Informative letter for the obstetrician | Once in the pre-conception period  Twice, if genotype analysis is performed |
|  | Biochemistry | serum Phe, Tyr, Phe/Tyr | Every 15 days |
|  | Biochemistry | Other amino acids | 1/month |
|  | Biochemistry | Folic acid, vitamin B12, full blood count, ferritin, vitamin D, Calcium,  Phosphorous, lipid profile, liver function, creatinine, serum protein  electrophoresis, serum glucose, thyroid function | Every 6 months |
| **Pregnancy** | Medical  evaluation | Physical examination  Reinforce information on type and timing of check-ups during pregnancy  Review pharmacological therapies for PKU | 1/month |
|  | Nutritional  evaluation | Weight, Height, BMI, Body composition  Review Food diary  Review Tolerance to Phe  Review lifestyle (e.g. physical activity, alcohol)  Review acceptance of medical formula (in terms of palatability and personal choice)  Review availability of hypoproteic foods and medical formula  Reinforce information on the type and timing of check-ups during pregnancy | 1/week |
|  | Genetic  counseling | Counseling on reproductive risk based on metabolic control  Reinforce information of the partner about PKU and risk of MPKUS  Renew information on the possibility for the partner to perform the mutational analysis of the PAH gene to assess his eventual carrier status  Renew information on the neonatal screening for PKU for all newborns in Italy  Update informative letter for the obstetrician | within 1 week from pregnancy notification and between 8-10 weeks of GA |
|  | Biochemistry | serum Phe, Tyr, Phe/Tyr | 1-2/week |
|  | Biochemistry | Other amino acids | 1/month |
|  | Biochemistry | folic acid, vitamin B12, full blood count, ferritin, vitamin D,  Calcium, Phosphorous, lipid profile, liver function, creatinine, serum protein  electrophoresis, serum glucose, thyroid function | At the beginning of the pregnancy and after 6 months. |

Abbreviations: BMI, body mass index; PKU, phenylketonuria; Phe, phenylalanine; Tyr, tyrosine; MPKUS, maternal phenylketonuria syndrome; PHA, phenylalanine hydroxylase; GA, gestational age.

**Supplementary File 2**

**Supplementary table S2.** Standard operating procedure for the management of unplanned pregnancies (UP) of women with PKU adopted by the Clinical Center

|  | **Assessment** | **Items analyzed/discussed** | **Note** |
| --- | --- | --- | --- |
| **First pregnancy**  **notification of a women with**  **PKU ON-DIET** | Medical evaluation | Physical examination  Provide information on the type and timing of check-ups during pregnancy  Review Pharmacological therapies for PKU (sapropterin, pegvaliase)  Provide information on the use of pharmacological therapies during pregnancy  Discuss the risk-benefits of pharmacological therapies on an individual basis | Within 24 hours from pregnancy notification |
|  | Nutritional  evaluation | Weight, Height, BMI, Body composition  Request of a 3-days food diary  Food interview  Review tolerance to Phe and delivery of a diet based on tolerance to Phe  Review lifestyle (e.g. physical activity, alcohol, etc)  Review availability of hypoproteic foods and medical formula  Review acceptance of medical formula (in terms of palatability and personal choice)  Provide information on the type and timing of check-ups during pregnancy | Within 24 hours from pregnancy notification |
|  | Genetic counseling | Counseling on reproductive risk based on metabolic control and GA (risk of MPKUS)  Review genotype analysis  Counselling on recurrence risk for future pregnancies  Information of the partner about PKU and the risk of MPKUS  Information on the possibility for the partner to perform the mutational analysis of the PAH gene to assess his eventual carrier status  Information on the neonatal screening for PKU for all newborns in Italy  Informative letter for the obstetrician | Within 7 days from pregnancy notification |
|  | Biochemistry | serum Phe, Tyr, Phe/Tyr  Other amino acids | Within 24 hours from pregnancy notification |
|  | Biochemistry | folic acid, vitamin B12, full blood count, ferritin, vitamin D, Calcium, Phosphorous, lipid profile, liver function, creatinine, serum protein electrophoresis, serum glucose, thyroid function | Within 7 days from pregnancy notification |
| **First pregnancy notification of a women with**  **PKU OFF-DIET** | Medical evaluation | Physical examination  Provide information on the type and timing of check-ups during pregnancy  Review Pharmacological therapies for PKU (sapropterin, pegvaliase)  Provide information on the use of pharmacological therapies during pregnancy  Discuss the risk-benefits of pharmacological therapies on an individual basis | Within 24 hours from pregnancy notification |
|  | Nutritional  evaluation | Weight, Height, BMI, Body composition  Review availability of hypoproteic foods and medical formula  If unavailable, provide medical prescription and free samples of hypoproteic foods and medical formula  Food interview  Request of a 3-days food diary, reinforcing education  Review tolerance to Phe and delivery of a diet based of historical tolerance (if available)  If tolerance is unknown, initial Phe intake is calculated according to serum Phe level  Review of lifestyle (e.g. physical activity, alcohol, etc.)  Review of acceptance of medical formula (in terms of palatability and personal choice)  Provide information on the type and timing of check-ups during pregnancy | Within 24 hours from pregnancy notification |
|  | Genetic counseling | Counseling on reproductive risk based on metabolic control and GA (risk of MPKUS)  Review genotype analysis  Counselling on recurrence risk for future pregnancies  Information of the partner about PKU and the risk of MPKUS  Information on the possibility for the partner to perform the mutational analysis of the PAH gene to assess his eventual carrier status  Information on the neonatal screening for PKU for all newborns in Italy  Informative letter for the obstetrician | Within 7 days from pregnancy notification |
|  | Biochemistry | Serum Phe, Tyr, Phe/Tyr  Other amino acids | Within 24 hours from pregnancy notification |
|  | Biochemistry | folic acid, vitamin B12, full blood count, ferritin, vitamin D, Calcium, Phosphorous, lipid profile, liver function, creatinine, serum protein electrophoresis, serum glucose, thyroid function | Within 7 days from pregnancy notification |
| **Pregnancy**  **follow-up** | Medical evaluation | Physical examination  Reinforce information on the type and timing of check-ups during pregnancy  Review pharmacological therapies for PKU | 1/ month |
|  | Nutritional  evaluation | Weight, Height, BMI, Body composition  Review availability of hypoproteic foods and medical formula  If still unavailable, continue to provide samples of hypoproteic foods and medical formula  Review Food diary  Review Tolerance to Phe  Review lifestyle (e.g., physical activity, alcohol)  Review acceptance of medical formula (in terms of palatability and personal choice)  Reinforce information on the type and timing of check-ups during pregnancy | 1/week |
|  | Genetic counselling | Counselling on reproductive risk based on metabolic control  Reinforce information of the partner about PKU and risk of MPKUS  Renew information on the possibility for the partner to perform the mutational analysis of the PAH gene to assess his eventual carrier status  Renew information on the neonatal screening for PKU for all newborns in Italy  Update informative letter for the obstetrician | Within 10th week of gestational age |
|  | Biochemistry | serum Phe, Tyr, Phe/Tyr | 1-2/week |
|  | Biochemistry | Other amino acids | 1/month |
|  | Biochemistry | Folic acid, vitamin B12, full blood count, ferritin, vitamin D, Calcium, Phosphorous, lipid profile, liver function, creatinine, serum protein electrophoresis, serum glucose, thyroid function | Every 3 months |

Abbreviations: BMI, body mass index; PKU, phenylketonuria; Phe, phenylalanine; Tyr, tyrosine; MPKUS, maternal phenylketonuria syndrome; PHA, phenylalanine hydroxylase; GA, gestational age.

***Supplementary File 3***

*Dietary management during pregnancy*

Protocols were adapted on a case-by-case basis:

- Assessment of caloric intake: ideal body weight (P.I., kg) multiplied by energy quotient (E.Q.=30-35 kcal);
- Normo-proteic dietary therapy low in phenylalanine (based on individual tolerance) in preparation; normo-protein dietary therapy, corrected in protein and low in phenylalanine, compatible with physiological status of pregnant women during pregnancy;
- Periodic caloric supplementation based on individual patients' increased needs and body mass index (BMI).

Based on the 4th revision of the LARN (Reference Intake Levels of Nutrients and Energy for the Italian Population) tables and the different individual caloric requirements, in pregnant women starting from underweight and normal weight condition (BMI<18.5 kg/m^2^ and 18.5≤IMC≥24.9 kg/m^2^, respectively) caloric additions were made from the second trimester; in overweight and obesity conditions, caloric additions were assessed according to the patients' lifestyle **[Table S3].** Caloric nutrient intake, i.e., carbohydrates, lipids, and protein, was divided into protein calories and non-protein calories. Protein calories were provided initially for an intake of 0.9 g/kg/day (in preparation of pregnancy) increased to 1.2 g/kg/day at the beginning of pregnancy. Non-protein calories were separated into 25-30% lipids and the remaining 50-60% carbohydrates. In the first trimester, the addition of protein to the diet was very limited but was gradually increased as the pregnancy progressed. From each pregnant woman's historical phenylalanine tolerance, a calculation was made of the maximum amount of natural protein, containing phenylalanine, that each could take from food. It was then calculated the remaining amount of protein to be taken through amino acid supplements considering that the total protein intake should provide the safe intake levels of individual protein requirements (FAO/WHO/UNU 2007) with an additional 40% L-amino acids, administered in the form of supplements **[1]**. To calculate the amount of amino acids to be supplemented daily, the formula used was as follows: {[ideal body weight (kg) × 0.9] - natural protein intake (g)} × 1.4 = dose (g).

This calculation includes an additional 20% L-amino acids to compensate for the so-called "essential amino acid score" and an additional 20% L-amino acids to optimize their effect on blood phenylalanine control. If inadequate doses of L-amino acids are administered, they become limiting for protein synthesis and metabolism shifts to catabolism. In protein catabolism, phenylalanine will not only remain unused but will also be released and its concentration will increase. Therefore, the administration of L-amino acids in the right amounts is crucial as it reduces the blood phenylalanine concentration. Protein supplements were made with medical formulas of powdered amino acids or liquid blends. The choice of supplement was not straightforward, and several palatability tests were performed to evaluate the most suitable one for each pregnant woman. Protein supplements from the second trimester onward involved the administration of amino acids alone in the form of microgranules. In all pregnancies, cereal-based and reduced-protein special medical purpose foods such as bread and pasta were prescribed; patients consumed replacement milk or alternatively rice milk (0.3 g protein per 100 g). In Italy there are no barriers to insurance coverage, and access to low-protein products and medical formulas is not restricted thanks to the disease exemption, which makes dietary therapy easier for patients who do not have economic possibilities to purchase the products. Daily folic acid intake was 400 µg/day both when preparing for pregnancy and during pregnancy. Where iron supplementation was needed, it was added. Because phenylalanine tolerance could increase during pregnancy, dietary amino acid intake was re-evaluated in each patient from the second trimester, considering blood values of phenylalanine and tyrosine and adherence to nutritional therapy. Folic acid was supplemented even though it was already present in medical formulas. Omega 3 (DHA) supplementation (200 mg/day) was carried out in patients up to the eighth month of pregnancy and discontinued the last month so as not to affect the clotting process at the time of delivery.

**Supplementary Table S3.** Dietary macronutrient composition for patients in pregnancy preparation and during the pregnancy, based on the latest LARN (Reference Intake Levels of Nutrients and Energy for the Italian Population) [2].

| **TOTAL CALORIES** | **P.I.x 30-35 kcal** |
| --- | --- |
| **PROTEIN CALORIES** |  |
| **Protein**  I trimester  II trimester  III trimester | **0.9 g/kg/day**  +1 g/day  +8 g/day  +26 g/day |
| **NON PROTEIN-CALORIES** |  |
| **Fats** | **25-30%** |
| Saturated | <10% |
| Monounsaturated | 10-12% |
| Polyunsaturated | 6-8% |
| **Carbohydrates** | **50-60%** |
| Complex | 40-50 |
| Simple | 8-10% |
| **FIBRES** | **At least 20 g/day** |
| **WATER SUPPLY** | **40 mL/kg/day** |
| **Calcium** | **1200 mg/day** |
| **Folic acid** | **+400 µg/day** |
| **DHA** | **+200 mg/day** |

**References**

1. van Spronsen, F.J.; van Wegberg, A.M.; Ahring, K.; Bélanger-Quintana, A; Blau, N.; Bosch, A.M.; Burlina, A.; Campistol, J.; Feillet, F.; Giżewska, M.; Huijbregts, S.C.; Kearney, S.; Leuzzi, V.; Maillot, F.; Muntau, A.C.; Trefz, F.K.; van Rijn, M.; Walter, J.H.; MacDonald, A. Key European guidelines for the diagnosis and management of patients with phenylketonuria. *Lancet Diabetes Endocrinol*. **2017** Sep;*5(9):*743-756. doi: 10.1016/S2213-8587(16)30320-5. Epub 2017 Jan 10. PMID: 28082082.
2. SINU, Italian Society of Human Nutrition. LARN - Reference Intake Levels of Nutrients and Energy for the Italian Population. IV Revision. Editorial coordination SINU-INRAN. Milan: SICS, 2014.
